# Correction: Molybdenum-doped iron oxide nanostructures synthesized *via* a chemical co-precipitation route for efficient dye degradation and antimicrobial performance: *in silico* molecular docking studies

**DOI:** 10.1039/d3ra90065g

**Published:** 2023-07-25

**Authors:** Tahira Shujah, Anum Shahzadi, Ali Haider, Muhammad Mustajab, Afsah Mobeen Haider, Anwar Ul-Hamid, Junaid Haider, Walid Nabgan, Muhammad Ikram

**Affiliations:** a Department of Physics, University of Central Punjab Lahore 54000 Punjab Pakistan; b Faculty of Pharmacy, The University of Lahore Lahore Pakistan; c Department of Clinical Medicine, Faculty of Veterinary and Animal Sciences, Muhammad Nawaz Shareef, University of Agriculture 66000 Multan Punjab Pakistan; d Solar Cell Applications Research Lab, Department of Physics, Government College, University Lahore Lahore 54000 Punjab Pakistan dr.muhammadikram@gcu.edu.pk; e Core Research Facilities, King Fahd University of Petroleum & Minerals Dhahran 31261 Saudi Arabia anwar@kfupm.edu.sa; f Tianjin Institute of Industrial Biotechnology, Chinese Academy of Sciences Tianjin 300308 China; g Departament d’Enginyeria Química, Universitat Rovira i Virgili 43007 Tarragona Spain wnabgan@gmail.com

## Abstract

Correction for ‘Molybdenum-doped iron oxide nanostructures synthesized *via* a chemical co-precipitation route for efficient dye degradation and antimicrobial performance: *in silico* molecular docking studies’ by Tahira Shujah *et al.*, *RSC Adv.*, 2022, **12**, 35177–35191, https://doi.org/10.1039/D2RA07238F.

The authors regret that some of the author affiliations were incorrectly shown in the original manuscript. The corrected list of affiliations is as shown above.

The Royal Society of Chemistry apologises for these errors and any consequent inconvenience to authors and readers.

## Supplementary Material

